# Characterization of acquired nutlin-3 resistant non-small cell lung cancer cells

**DOI:** 10.20517/cdr.2020.91

**Published:** 2021-03-19

**Authors:** Christophe Deben, Laurie Freire Boullosa, Andreas Domen, An Wouters, Bart Cuypers, Kris Laukens, Filip Lardon, Patrick Pauwels

**Affiliations:** ^1^Center for Oncological Research (CORE), Integrated Personalized & Precision Oncology Network (IPPON), University of Antwerp, Wilrijk 2610, Belgium.; ^2^Adrem Data Lab, Department of Computer Science, University of Antwerp, Antwerp 2020, Belgium.; ^3^Molecular Parasitology Unit, Department of Biomedical Sciences, Institute of Tropical Medicine, Antwerp 2000, Belgium.; ^4^Department of Pathology, Antwerp University Hospital, Edegem 2650, Belgium.

**Keywords:** p53, murine-double minute 2, non-small cell lung cancer, acquired resistance, nutlin-3

## Abstract

**Aim**: The purpose of this manuscript is to study the potential characteristics of acquired nutlin-3 resistant non-small cell lung cancer cells (NSCLC). Nutlin-3 is an inhibitor of the murine-double minute 2 protein, the main negative regulator of wild type p53, of which several derivatives are currently in clinical development.

**Methods**: A549 NSCLC cells were exposed to increasing concentrations of nutlin-3 for a period of 18 weeks. Monoclonal derivates were cultured, and the most resistance subclone was selected for whole transcriptome analysis. Gene set enrichment analysis was performed on differentially expressed genes between A549 nutlin-3 resistant cancer cells and the parental A549 p53 wild type cancer cells. Relevant findings were validated at the gene, protein and/or functional level.

**Results**: All nutlin-3 resistant subclones acquired mutations in the *TP53* gene, resulting in overexpression of the mutant p53 protein. The most resistant subclone was enriched for genes related to epithelial to mesenchymal transition (EMT), resulting in increased migratory and invasive potential. Furthermore, these cells were enriched in genes related to inflammation, tissue remodelling, and angiogenesis. Importantly, expression of several immune checkpoints, including PD-L1 and PD-L2, was significantly upregulated, and cisplatin-induced cell death was reduced.

**Conclusion**: Transcriptome analysis of a highly nutlin-3 resistant A549 subclone shows the relevance of studying (1) resistance to standard of care chemotherapy; (2) secretion of immunomodulating chemo- and cytokines; (3) immune checkpoint expression; and (4) EMT and invasion in nutlin-3 resistant cancer cells in addition to acquired mutations in the *TP53* gene.

## Introduction

The p53 protein is considered to be “the guardian of the genome” and protects healthy cells against malignant transformation. Consequently, its function is often disturbed in cancer cells by either inactivating or gain-of-function mutations in the *TP53* gene^[1]^. Alternatively, wild type p53 levels can be suppressed by overexpression of p53’s main negative regulator, i.e., murine double minute 2 (MDM2) protein^[2,3]^. For the latter, selective inhibitors of the interaction between MDM2 and p53 have been developed with nutlin-3 being the first-in-class small molecule^[4]^. Increased levels of wild type p53 following nutlin-3 treatment lead to activation of downstream effector pathways related to cell cycle arrest, senescence, or apoptosis. This activation can be further enforced in combination with stress-inducing agents like cisplatin, inducing DNA damage and leading to synergistic anti-cancer effects in wild type p53 cancer cells^[5]^.

Resistance to targeted therapies remains a major problem in the clinic and predictive biomarkers for therapy response are crucial. For nutlin-3, or its derivates currently in clinical development (RG7112 and RG7388), it is clear that wild type p53 is an important prerequisite for response^[6]^. However, little is known about acquired resistance mechanisms and the phenotype of these resistant cancer cells. Therefore, we generated nutlin-3 resistant A549 non-small cell lung cancer cells (NSCLC). Following monoclonal expansion of these resistant cells, we selected the most resistant subclone for in-depth analysis of its characteristics by means of whole transcriptome analysis and provide an overview of characteristics worth studying in nutlin-3 resistant cancer cells.

## Methods

### Cell lines and cell culture

**Figure 1:**
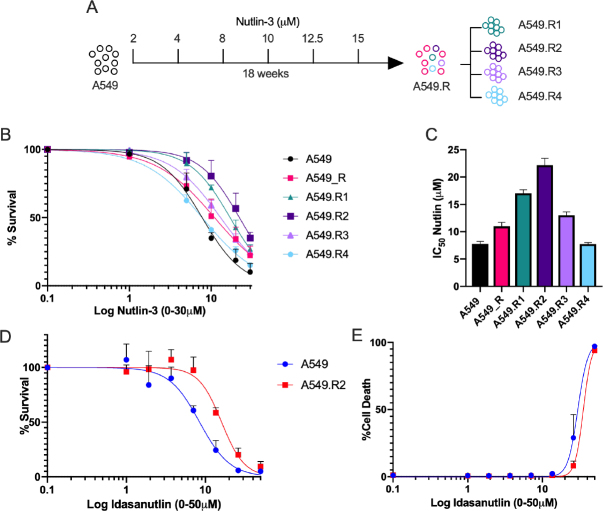
Acquired nutlin-3 resistance. A549 cells were exposed to increasing concentrations of nutlin-3 for a period of 18 weeks. From these nutlin-3 exposed A549 cells, four monoclonal subclones were cultured (A); dose response curve for nutlin-3 of A549 cells, the parental resistant A549.R cells and four monoclonal subclones using the SRB assay (A549.R1-R4) (B); corresponding nutlin-3 IC_50_ values (C); percentage survival (D); and percentage cell death of A549 and A549.R2 cells treated with idasanutlin (0-50 µmol/L) for 96 h determined by the IncuCyte ZOOM cytotoxicity assay (E). Data presented as mean +/- SD of at least 3 biological replicates

The NSCLC cell lines A549 (p53 wild type) and NCI-H1975 (p53 mutant R273H) were obtained from ATCC. Cells were grown as monolayers and maintained in exponential growth in 5% CO_2_/95% air in a humidified incubator at 37 °C. A549 and NCI-H1975 cells were, respectively, cultured in DMEM or RPMI (Life Technologies) supplemented with 10% fetal bovine serum (FBS, Life Technologies), 1% penicillin/streptomycin (Life technologies) and 2 mmol/L L-glutamine (Life technologies). A549 cells were exposed to increasing concentrations of nutlin-3 (Tocris) over a period of 18 weeks [Figure 1A, Supplementary Table 1] to generate nutlin-3 resistant cancer (A549.R) cells. After 18 weeks, cells were diluted to 1000 cells/mL and 1 µL was added to a 96-well plate to generate monoclonal subclones. Four subclones (A549.R1 - R4) were successfully generated. A549.R2 was identified as the most resistant subclone and used for further characterization of nutlin-3 resistance mechanisms.

A separate batch of A549 and A549.R2 cells were stably transduced with the Incucyte® Nuclight red lentivirus reagent with a puromycin selection gene to allow cell tracking on the IncuCyte ZOOM system (Sartorius). All cell lines were confirmed as mycoplasma-free using the MycoAlert Mycoplasma Detection Kit.

### Sulforhodamine B cytotoxicity assay

A colometric sulforhodamine B (SRB) assay was used to measure treatment-induced cytotoxicity to nutlin-3. 1500 cells/well were plated and allowed to settle overnight. Afterwards they were exposed to 0-30 µmol/L nutlin-3 for 72 h. Cell monolayers were then fixed with 10% trichloroacetic acid for 1 h at 4 °C and stained with 100 µL 0.1% SRB, as previously described^[7]^. Dose response curves were plotted and corresponding IC_50_ values (i.e., drug concentrations causing 50% growth inhibition) were calculated using GraphPad Prism software.

### IncuCyte ZOOM cytotoxicity assay

Nuclight red transduced A549 and A549.R2 cells were seeded at a concentration of 500 cells/well in a 384-well plate and allowed to settle overnight. A 7-point titration of idasanutlin (0-50 µmol/L, Tocris, DMSO), APR-246 (0-50 µmol/L, Tocris, DMSO), cisplatin (0-20 µmol/L, Tocris, 0.3%Tween20 in 0.9%NaCl) and 75 nM cytotox green reagent (Sartorius, DMSO) was added using the Tecan D300e digital drug dispenser. After 96 hours the plate was scanned with the IncuCyte ZOOM and % survival was determined with the formula:

**Figure:**
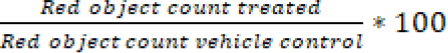


and % cell death with the formula:

**Figure:**
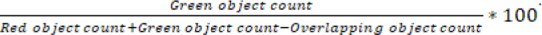


Dose response curves were plotted with GraphPad Prism software.

### IncuCyte ZOOM scratch wound assay

Nuclight red transduced A549 and A549.R2 cells were seeded at a concentration of 20.000 cells/well in an ImageLock 96-well plate (Sartorius) and allowed to settle overnight. Scratch wounds were made with the WoundMaker tool according to the manufacturer’s instruction. For invasion, scratched cells were overlayed with 6 mg/mL Matrigel matrix. Cell migration and invasion was monitored every 2 h using the IncuCyte Zoom system and analysed using the IncuCyte Scratch Wound Analysis Software module.

### Whole transcriptome analysis

Total RNA was isolated from 3 biological replicates of the parental A549 and nutlin-3 resistant A549.R2 cell lines using the Qiagen RNA Plus Mini Kit, according to the manufacturer’s instructions. RNA concentration and purity were checked using the NanoDrop ND-1000 (ThermoFisher). Samples were frozen at -80 °C and sent to BGI Genomics for quality control and transcriptome sequencing (oligo dT, non-stranded DNBseq platform, PE150 30M reads/9Gb). Raw sequencing reads were aligned to the human reference genome v38, and per gene read counts generated with STAR v2.7.2b^[8]^. Differential expression analysis was carried out in R with the DEseq2 package^[9]^. Volcano plots were generated with EnhancedVolcano in R. Preranked Gene Set Enrichment Analysis (GSEA) was performed using the www.webgestalt.org analysis tool kit v2019^[10]^ for WikiPathwaysCancer and KEGG databases and the Hallmark^[11]^ and Oncogenic Signatures gene sets downloaded from the Molecular Signatures Database v7.1 database^[12]^. Gene sets with a false discovery rate (FDR) ≤ 0.05 and *P*-value ≤ 0.05 were considered.

### Next-generation sequencing

DNA from A549 and its isogenic nutlin-3 resistant subclones was isolated using the GenElute Blood Genomic DNA kit (Sigma Aldrich) and DNA concentration and purity were checked using the NanoDrop ND-1000.

*TP53* NGS was performed using the *TP53* MASTR^TM^ with MID for Illumina Miseq kit (Multiplicom) according to the manufacturer’s instructions. Sequencing was performed on a MiSeq system using the MiSeq Reagent Kit v2 (500 cycles, Illumina). Using an in-house annotation and filtering tool, VariantDB, single nucleotide variants were annotated to the *TP53* NM_000546 transcript file^[13]^. SNVs present in the dbSNP137 and 1000 Genomes Project databases were identified as single nucleotide polymorphisms and excluded. The reads were visualised using the Integrative Genomics Viewer software (IGV, version 2.3.67) and aligned to the human reference genome (hg19, NCBI build 37). Using the MUT-TP53 2.0 tool, inactivating *TP53* mutations were identified^[14]^.

### Western blot

Cells were lysed in lysis buffer (10 mmol/L TrisHCl, 400 mmol/L NaCl, 1 mM EDTA, 0.1% NP40 and protease inhibitor). After centrifugation (10 min, 13 000 rpm, 4 °C), cleared lysates containing the isolated proteins were harvested and kept at -80 °C. Protein concentrations were determined using the Pierce BCA protein kit, according to the manufacturer’s instructions. Western blot was performed as described previously^[15]^. Blocking, primary and secondary antibody incubation was performed using the SNAP id 2.0 protein detection system, according to the manufacturer’s instructions. Membranes were incubated with rabbit anti-p53 (1:1000, Cell Signalling #9282) and mouse anti-b-actin (1:2500, Sigma-Aldrich). Goat anti-rabbit (1:10 000, Licor IRDye 800CW) and goat anti-mouse (1:10 000, Licor IRDye 680RD) fluorescently labelled secondary antibodies were used. Fluorescent detection was performed using the Odyssey imaging system (Li-Cor). Image Studio Lite software (Li-Cor) was used to perform pixel quantification of the images. Normalization against the internal actin control was performed for each sample.

## Results

### Acquired nutlin-3 resistance

**Figure 2:**
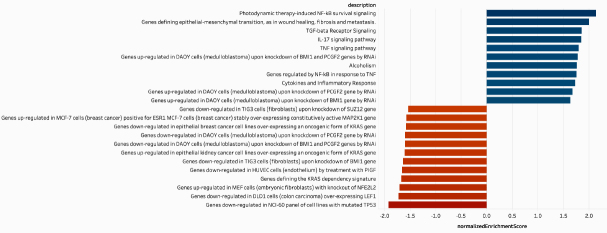
Gene set enrichment analysis of A549.R2 nutlin-3 resistant cells compared to A549 cells. Normalized enrichment scores (NES) of gene expression signatures with a FDR ≤ 0.05 and *P*-value ≤ 0.05 are presented from the WikiPathwaysCancer, KEGG, Hallmark and Oncogenic Signatures gene sets. A detailed overview and gene set names are presented in Supplementary Table 2.

Long term exposure to nutlin-3 resulted in a slight decrease in sensitivity of the A549 *vs*. A549.R cells (IC_50_: 7.7 µmol/L *vs*. 11.0 µmol/L; Figure 1B and C). Within the monoclonal subclones, a strong variability in sensitivity was observed, with the A549.R2 subclone being the most resistant clone (IC_50_: 22.2 µmol/L; Figure 1B and C). Reduced sensitivity is also retained for idasanutlin (RG7388, IC_50_: 8.45 µmol/L *vs*. 16.04 µmol/L), a more potent second-generation nutlin-3 currently in clinical development, which induced a predominant cytostatic response [Figure 1D and E]. We selected the A549.R2 clone for further downstream analysis of the molecular characteristics of acquired nutlin-3 resistance by means of transcriptome analysis. Supplementary Figure 1 represents the most differentially expressed genes between A549.R2 and the parental A549 cells. This differentially expressed gene sets was pre-ranked and subjected to GSEA enrichment analysis and the top gene signatures are presented in Figure 2 and Supplementary Table 2 (FDR ≤ 0.05; *P*-value ≤ 0.05).

### Acquired TP53 mutations

**Figure 3:**
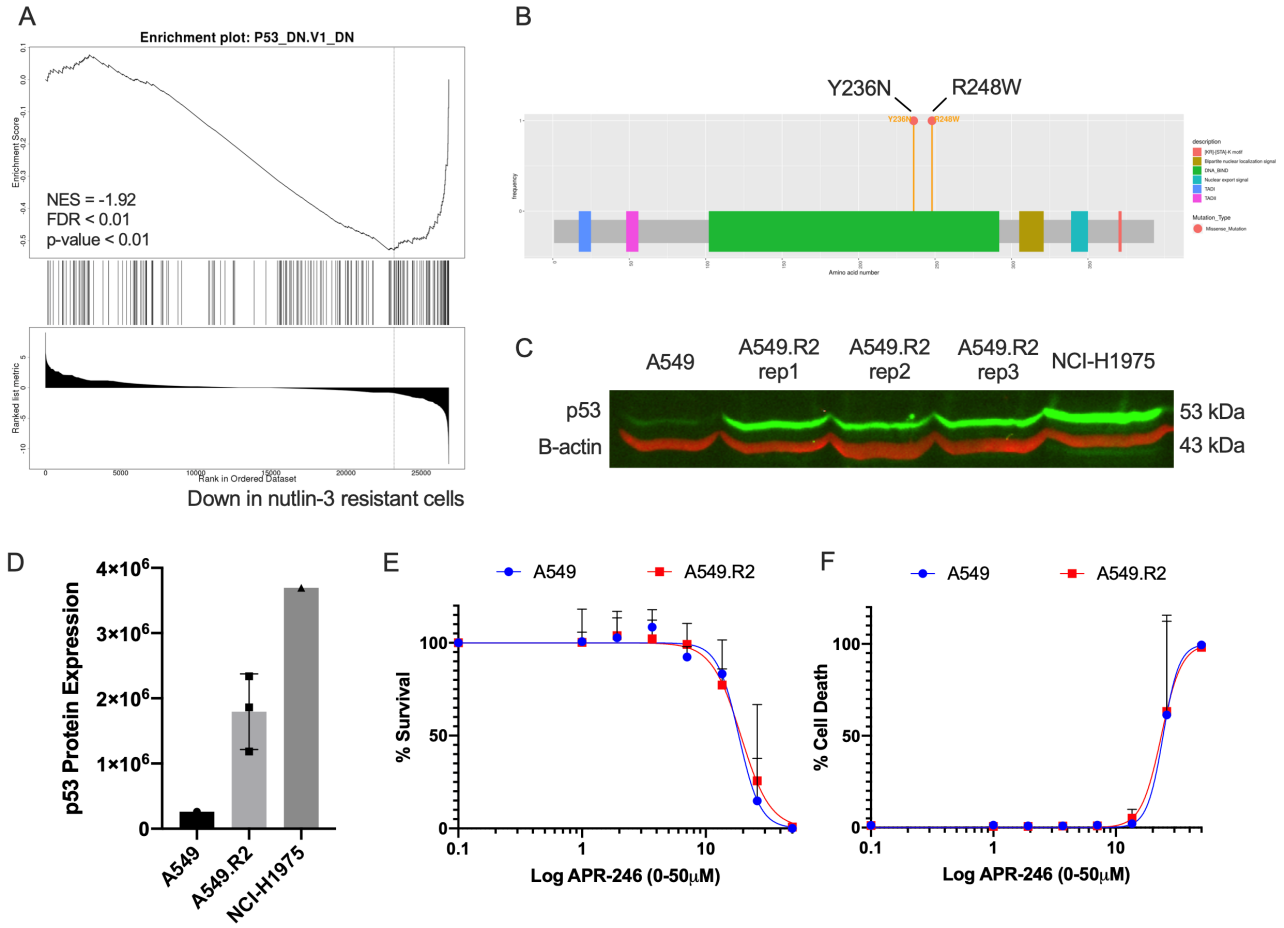
Acquired *TP53* mutations. Genes downregulated in NCI-60 cell line panel with mutated p53 (gene set: M2697) (A); mutations in the *TP53* gene detected in the A549.R2 cell line (B); Western Blot analysis of p53 protein expression (C) and fluorescent intensity normalized to b-actin (D); percentage survival (E) and percentage cell death (F) of A549 and A549.R2 cells treated with APR-246 (0-50 µmol/L) for 96 h determined by the IncuCyte ZOOM cytotoxicity assay. Data presented as mean +/- SD of at least 3 biological replicates

GSEA analysis indicates that A549.R2 cells have a gene signature related to cell lines with mutated p53 (gene set: M2697, Figure 3A). Targeted NGS further confirmed the presence of two heterogenous missense mutations, Y236N and R248W, in the A549.R2 cells [Figure 3B, Supplementary Table 3]. No mutations were detected in the parental A549 cell line, and similar mutations were detected in the other monoclonal subclones A549.R1 (Y236N), A549.R3 (Y236N, R248W) and A549.R4 (Y236N) [Supplementary Table 3]. At the protein level, p53 was overexpressed in the A549.R2 cell line, which is often observed in mutant p53 cancer cells (like NCI-H1975) due to the disturbance of the MDM2 regulated negative feedback loop [Figure 3C and D]. Finally, we determined if the presence of these TP53 mutations could sensitize A549.R2 cancer cells to APR-246, a reactivator of mutant p53 and/or inducer of oxidative stress. However, we did not observe any difference in cell survival [Figure 3E] or cell death [Figure 3F] between the wild type A549 and mutant A549.R2 cell lines.

### Epithelial to mesenchymal transition

**Figure 4:**
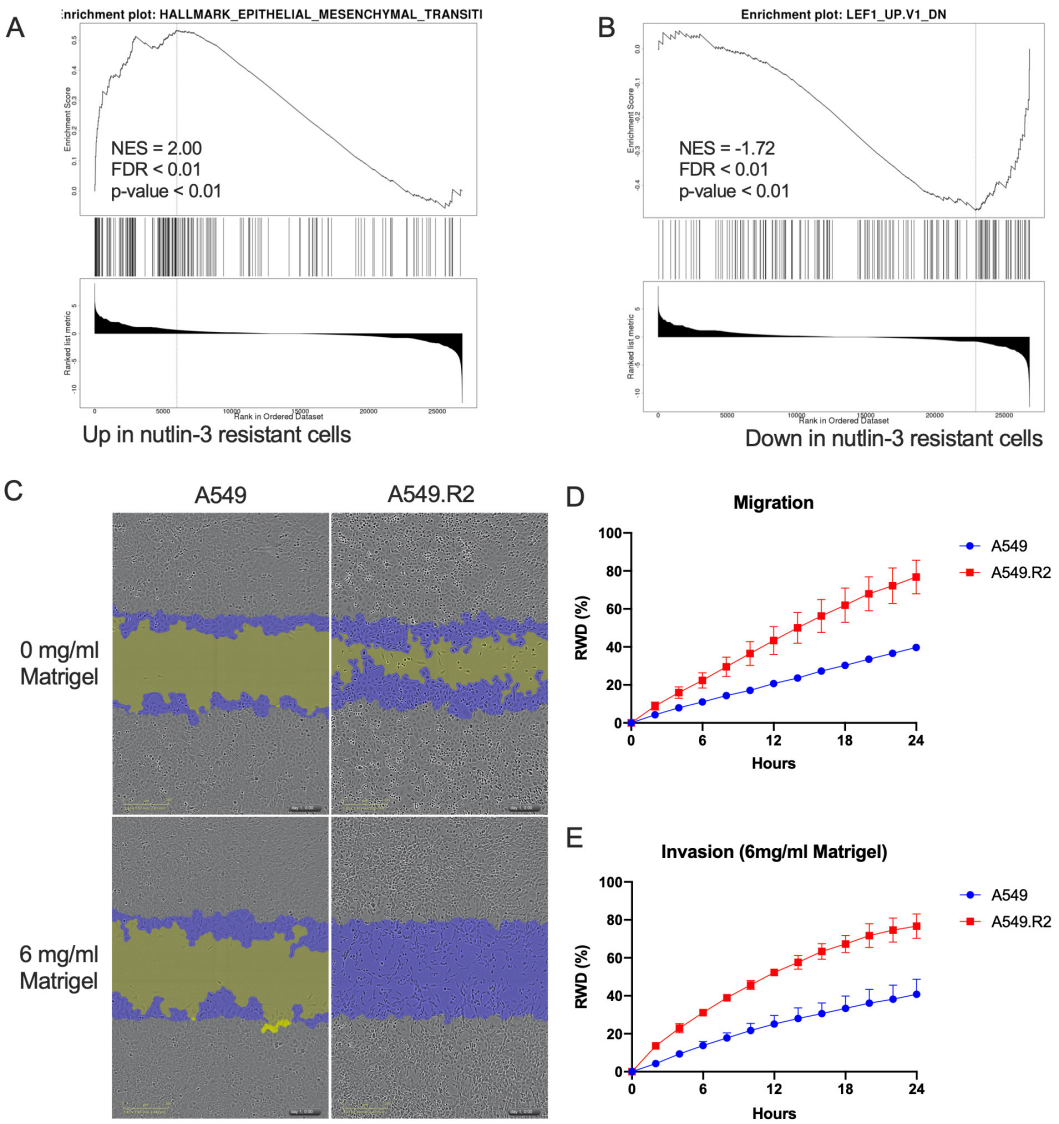
Epithelial to mesenchymal transition (EMT). Genes defining EMT (gene set M5930) (A); genes down-regulated in DLD1 cells (colon carcinoma) over-expressing LEF1 (gene set: M2903) (B); representative images (24 h) of a scratch wound migration (0 mg/mL Matrigel) and invasion (6 mg/mL Matrigel) assay of the nutlin-3 resistant A549.R2 cell line and parental A549 cell line (C); real-time migration (D) and invasion (E). RWD: relative wound density presented as mean +/- SD of 3 biological replicates

A549.R2 cells have an increased enrichment of an EMT-like expression signature (gene set: M5930, Figure 4A). EMT is potentially mediated by activation of LEF1, a transcription factor which regulates transcription of hallmark EMT effectors, since A549.R2 cells are enriched for genes downregulated in LEF1 overexpressing cells (Figure 4B; gene set: M2903). Consistent with a mesenchymal-like phenotype, A549.R2 cells have an increased migratory and invasive potential compared to the parental A549 cells [Figure 4C-E].

### Immunologic signatures

**Figure 5:**
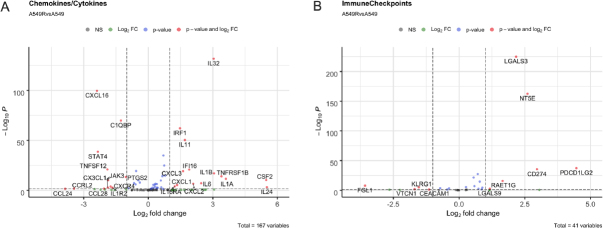
Volcano plot of differentially expressed genes in nutlin-3 resistant A549.R2 cells versus parental A549 cells. Chemokine and cytokine gene set derived from the Nanostring nCounter^®^ PanCancer Immune Profiling Panel (A); immune checkpoints gene set based on literature (B). Log2 fold change cut-off ≤ -0.5 / ≥ 0.5, *P*-value cut-off ≤ 0.05

A549.R2 cells are enriched in genes related to NF-kB signalling (gene set WP3617, Supplementary Figure 2), IL-17 signalling (gene set hsa04657, Supplementary Figure 3), TNF signalling (gene set hsa04668, Supplementary Figure 4) and cytokines and inflammatory response (gene set WP530, Supplementary Figure 5). The affected genes are mostly related to inflammation, tissue remodelling, angiogenesis, and consist mainly of chemo- and cytokines [Supplementary Figures 2-5]. When focusing on an independent chemo- and cytokine gene set, we also observed downregulated genes as presented in Figure 5A. In addition, we observed an increased expression of several immune checkpoints including PD-L1 (CD274), PD-L2 (PDCD1LG2), CD73 (NT5E) and galectin-3 (LGALS3) as presented in Figure 5B.

### Oncologic signatures

**Figure 6:**
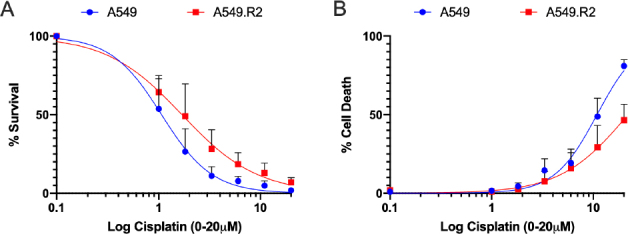
Cisplatin-induced cell death is reduced in A549.R2 cells. Percentage survival (A) and percentage cell death (B) of A549 and A549.R2 cells treated with cisplatin (0-50 µmol/L) for 96 hours determined by the IncuCyte ZOOM cytotoxicity assay. Data presented as mean +/- SD of at least 3 biological replicates

A549.R2 cells showed a reduced expression of genes defining KRAS dependency (gene set M2851; Supplementary Figure 6B) or related to overexpression of oncogenic KRAS (gene set M2892; Supplementary Figure 6B) compared to the parental A549 cancer cells (KRAS mutation p.G12S c.34G>A). Next, A549.R2 cells are enriched in gene sets related to knockdown of BMI-1 (gene sets: M2730, M2775, M2782, M2779; Supplementary Figure 6A), an oncogene related to chemoresistance^[16]^, including cisplatin^[17,18]^. Therefore, we determined the sensitivity of A549.R2 and A549 cancer cells for cisplatin. Only a small difference in survival was observed (IC_50_: 1.07 µmol/L *vs*. 1.69 µmol/L), but the induction of cell death was strongly reduced in the A549.R2 cells (EC_50_: 10.88 µmol/L *vs*. 21.93 µmol/L) [Figure 6A and B]. Finally, A549.R2 cells showed gene expression characteristics related to NFE2L2 activation (gene set: M2903; Supplementary Figure 6C), PCGF2 knockdown (gene sets: M2775, M2783, M2784; Supplementary Figure 6A), activation of TGF-b receptor signalling (gene set WP560, Supplementary Figure 7), SUZ12 knockdown (gene set: M2740; Supplementary Figure 6D) and MEK signalling (gene set: M2725; Supplementary Figure 6E).

## Discussion

In this study, we successfully generated nutlin-3 resistant A549 NSCLC cancer cells to study the characteristics of acquired resistance. Consistent with previous studies^[19,20]^, we observed that nutlin-3 selects for mutant p53 cancer cells since all subclones harboured at least one *TP53* mutation. Michaelis *et al.*^[20]^ suggest that nutlin-3 induces *de novo* p53 mutations due to the high variability in mutations they detected in their sublines. Similarly, Aziz *et al.*^[21]^ report that four different p53 mutations were obtained in four separate experiments in which nutlin-3 resistant SJSA-1 cell were generated. Although we only observed two different mutations (R248W and Y236N), the fact that we obtained two subclones with a double heterogenous mutation also suggest that these mutations are *de novo* mutations that can accumulate rather than a selection of pre-existing mutant p53 A549 cells since all clones have the same Y236N mutation and two clones acquired an additional R248W mutation. This is further supported by the fact that no mutations were detected in the parental A549 cells following deep-sequencing analysis. Finally, Skalniak *et al.*^[22]^ report similar findings for Idasanutlin which generates p53-mutated drug-resistant subpopulations.

Despite the presence of p53 mutations in all the resistant subclones, a strong variability in nutlin-3 sensitivity was observed. Prolonged exposure to increasing concentrations of nutlin-3 would further select for the most resistant clones. Therefore, we selected the most resistant subclone A549.R2 for in-depth characterization by whole-transcriptome analysis to provide novel insights into the characteristics of nutlin-3 resistant cancer cells besides acquisition of p53 mutations and multidrug resistance as previously described^[20-22]^. Although our results cannot be generalized to all nutlin-3 resistant cells, our findings highlight interesting adaptations of cancer cells following continuous exposure to nutlin-3 that could be considered in other studies.

Acquiring a *TP53* mutation did not sensitize A549.R2 cells for treatment with APR-246 (PRIMA-1^Met^), a first in class reactivator of mutant p53^[23]^. It is important to note that increasing evidence suggests that the main mechanism of action of this compound is the induction of oxidative stress through its influence on thioredoxin and glutathione antioxidant systems and that restoring wild type p53 conformation is limited to specific mutations^[24,25]^. Nevertheless, this also suggests that A549.R2 cells are not sensitized to antioxidant inhibitors. In line with this observation, genes upregulated in cells with knockout of NFE2L2 were downregulated in the A549.R2 cells showing evidence of increased activity of the Nrf2 transcription factor, a master regulator of the cellular antioxidant response^[26]^.

Several gene sets related to BMI-1 knockdown were enriched in the A549.R2 cells. BMI-1 plays a vital role in cancer cell proliferation, invasion/metastasis, chemo-sensitivity and patient survival^[16]^. Wang *et al.*^[18]^ found that BMI-1 gene silencing can reduce intracellular glutathione levels in ovarian cancer cells, thus decreasing their resistance to the chemotherapeutic drug cisplatin^[17,18]^. However, we observed that A549.R2 cells were more resistant to cisplatin treatment, manifested as a reduced induction of cell death, consistent with the findings of Michaelis *et al.*^[20]^ in acquired nutlin-3 resistant neuroblastoma cells. Cisplatin acts by inducing DNA damage, thereby triggering the p53-regulated DNA-damage response. Our group recently showed that nutlin-3 synergistically enhances the cytotoxic effects of cisplatin through stimulation of wild type p53 leading to increased cell cycle arrest and apoptosis^[5]^. Consequently, loss of wild type p53 functions due to acquired mutations can disturb this response leading to increased resistance.

Further related to NSCLC standard of care therapy, A549.R2 cells acquired several characteristics that can modulate the tumour microenvironment, which could impact the response to standard of care immunotherapies like anti-PD-1/PD-L1 blockers. Firstly, gene expression of several matrix metalloproteinases (MMPs) was upregulated as indicated by pathway analysis of enriched gene sets WP3617 and hsa04657. These MMPs play a role in tissue remodelling and neo-angiogenesis which could affect treatment response to immunotherapy since combination strategies of immunotherapy and antiangiogenic compounds show promise for synergistic treatment of advanced NSCLC^[27-29]^. Secondly, the expression of a variety of chemo- and cytokines was affected in the A549.R2 cells which includes genes related to leukocyte recruitment (CXCL1-3) and activation (CSF1-2) and Th2 response (IL-5, IL-13) as indicated by the pathway analysis of gene sets hsa04668 and hsa04675, respectively. IL-32 was the most significantly upregulated cytokine, which has been shown to play a crucial role in tumour initiation, proliferation and maintenance^[30]^. Thirdly, we observed an increased expression of several immune checkpoints, including PD-L1 (CD274), PD-L2 (PDCD1LG2), CD73 (NT5E), and galectin-3 (LGALS3) in the nutlin-3 resistant cancer cells.

Finally, whole transcriptome analysis showed that nutlin-3 resistant cells acquire EMT-like properties. EMT-related transcription factors (Snail, Slug and ZEB1/2) suppress the expression of epithelial markers such as E-cadherin, while increasing the expression of mesenchymal markers N-cadherin and fibronectin^[31]^. An important characteristic of these mesenchymal-like cells is their increased motility which was confirmed by the increased migration and invasion of A549.R2 cells^[32]^. LEF1 is another transcription factor with an important role in EMT by activating transcription of hallmark EMT effectors such as N-cadherin, Vimentin and Snail^[33]^. A549.R2 were enriched in genes related to overexpression of LEF1, indicating that EMT is induced through induction of LEF1, although this needs to be confirmed at the protein and functional level.

In conclusion, transcriptome analysis of a highly nutlin-3 resistant A549 subclone shows the relevance of studying (1) resistance to standard of care chemotherapy; (2) secretion of immunomodulating chemo- and cytokines; (3) immune checkpoint expression; and (4) EMT and invasion in nutlin-3 resistant cancer cells in addition to acquired mutations in the *TP53* gene.

## Declarations

### Availability of data and materials

RNA-sequencing data will be deposited into data repositories upon acceptance of the manuscript.

### Copyright

© The Author(s) 2021.
